# GAS5 silencing attenuates hypoxia‐induced cardiomyocytes injury by targeting miR‐21/PTEN

**DOI:** 10.1002/iid3.945

**Published:** 2023-07-27

**Authors:** Qianli Wang, Zan Xie

**Affiliations:** ^1^ Cardiovascular Surgery Intensive Care Unit the Affiliated Yantai Yuhuangding Hospital of Qingdao University Yantai Shandong P.R. China; ^2^ Department of Cardiology the Affiliated Yantai Yuhuangding Hospital of Qingdao University Yantai Shandong P.R. China

**Keywords:** cardiomyocyte, cell injury, GAS5, hypoxia, MiR‐21

## Abstract

**Introduction:**

Myocardial hypoxia is an important factor causing myocardial infarction (MI). Interestingly, many unknown factors in the molecular mechanism of MI remain unclear. Our study explored the role of lncRNA growth arrest‐specific 5 (GAS5) in cell injury under hypoxia.

**Methods:**

AS5 expression was assessed in MI and human cardiomyocytes under hypoxia through RT‐qPCR assay. Methyl thiazolyl tetrazolium assay, flow cytometry assay, and transwell assay was carried out for cell viability, cell apoptosis, cell migration, and invasion, respectively. The regulatory target of GAS5 was explored through a dual‐luciferase reporter assay.

**Results:**

Our findings indicated that the upregulation of GAS5 was related to hypoxia. Downregulation of GAS5 expression could decrease hypoxia‐induced cell apoptosis and increase cell migration and invasion. Moreover, GAS 5 targeted miR‐21, which regulated the phosphatase and tension homology deleted on chromosome ten gene (PTEN) expression. Furthermore, the knockdown of miR‐21 eliminated the effect of GAS5 silencing on cell injury.

**Conclusion:**

These results indicated that lncRNA GAS5 silencing decreased cardiomyocyte injury by hypoxia‐induced through regulating miR‐21/PTEN.

## Graphical abstract

Our results indicate that GAS5 can regulate the process of myocardial infarction (MI) by targeting the miR‐21/PTEN axis. Therefore, both GAS5 and miR‐21/PTEN may become key targets for the treatment of MI in the future.

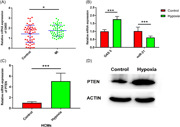

## 1 INTRODUCTION

Myocardial infarction (MI) occurs when blood flow to a part of the heart is reduced or stopped. MI damage to the myocardium has become one of the leading causes of cardiac morbidity and death.^1^ One of the most significant features of MI is the death of cardiomyocytes due to long‐term ischemia. Therefore, cardiomyocyte death is directly related to the occurrence and development of myocardial infarction blood.^2^ The consequences of ischemic injury are hypoxia, malnutrition, and finally apoptosis.^3^, ^4^ Therefore, the exact mechanism of MI remains to be studied in detail.

Long noncoding RNA is a transcript that can not encode proteins, including more than 200 nucleotides.^5^ Regardless of whether the transcribed lncRNA is in the nucleus or the cytoplasm, it can interact with nucleic acids or proteins to activate or inhibit downstream signaling molecules and affect the expression of transcription factors.^6^ LncRNAs have been reported to be involved in the regulation of physiological processes in different organisms, such as inflammation, DNA damage, and apoptosis.^7^ Furthermore, lncRNA is also involved in the development of ischemic heart disease, such as lncRNA CARL/miR539/PHB2 can provoke mitochondrial fission and apoptosis.^8^ Growth arrest‐specific 5 (GAS5) is a lncRNA that is closely associated with the regulation of malignant tumors. The expression of GAS5 is downregulated in renal cell carcinoma, and its downregulation has been suggested to serve an oncogenic role.^9^ Also, GAS5 expression is negatively correlated with the malignancy of cervical cancer, and its overexpression has been shown to reduce cell viability and increase apoptosis in cervical cancer cells.^10^ Furthermore, GAS5 has been reported to be involved in cell proliferation, invasion, metastasis, apoptosis, epithelial‐mesenchymal transition, and drug resistance through various molecular mechanisms.^11^ LncRNA GAS5 is associated with cardiac diseases. For example, GAS5 can modulate fibroblast and fibrogenesis through TGF‐β/Smad3 signaling.^12^ Add Wu et al. proved that downregulation of GAS5 ameliorates myocardial I/R injury via the miR‐335/ROCK1/AKT/GSK‐3β axis.^13^ In addition, lncRNA GAS5 has been reported to be involved in other biological processes, such as inflammation and apoptosis.^14^, ^15^, ^16^ As a type of noncoding small RNA, microRNA activates or inhibits the expression of target genes by binding to the 3’‐UTR sequences of target genes.^17^ Meanwhile, miRNAs also regulate the proliferative capacity, apoptosis rate, differentiation capacity of cells, and biological development process. Of course, MiRNA is also involved in regulating the process of hypoxic cardiomyocytes.

In our study, we aimed to investigate the function of GAS5 in hypoxia‐mediated cell injury. Firstly, we assessed GAS5 expression in hypoxia‐mediated cell injury. Secondly, We predicted and confirmed that GAS5 targeted miR‐21, which regulated PTEN expression. Overall, our results revealed that GAS5 attenuated hypoxia‐ induced cell injury in cardiomyocytes.

## 2 MATERIALS AND METHODS

### 2.1 Clincial sample

We collected peripheral blood samples from patients with MI. The inclusion and exclusion criteria for patients are: (1) The main clinical symptoms are persistent chest pain and chest tightness, which are not easily relieved by common drugs, such as Nitroglycerin, Suxiao Jiuxin Pill, etc; (2) The dynamic evolution of electrocardiogram, such as ST‐segment elevation, T‐wave inversion, and pathological Q‐wave formation; (3) Abnormal myocardial zymogram: myocardial zymogram examination includes Myoglobin, Troponin, and Creatine kinase isoenzymes. (4) If the above indicators are normal and coronary angiography is performed, if the stenosis is less than 50%, the possibility of MI can be ruled out. All operations have passed the review of the Yuhuangding Hospital's ethics committee.

### 2.2 Hypoxia treatment

Human cardiomyocyte primary cells (HCMs, Promocell, cat# C‐12810) were cultured in Dulbecco's modified eagle medium (DMEM, Gibco; Thermo Fisher Scientific, Inc.), including 10% FBS (Gibco), 100U/mL penicillin and 100U/ml streptomycin at 37°C, 5% CO_2_. To generate a model of cellular hypoxia, the cells were placed in an incubator with 94% N_2_, 5%CO_2_, and 1% O_2_, and cultured for 24 h, then the hypoxia‐treated cells were harvested for further assays.

### 2.3 MicroRNAs, cell transfections

The miRNAs including miR‐21 mimics, the corresponding mimics negative control and miR‐21 inhibitor were purchased from GenePharma. GAS5 siRNA (forward: 5’‐CACUCUGAGUGGGACAAGCUCUUCA−3’ and reserve: 5’‐UGAAG AGCUUGUCCCACUCAGAGUG−3’) and control siRNA (forward: 5’‐CACGAGUG GGUAACACUCGUCUUCA−3’ and reserve: 5’‐UGAAGACGAGUGUUACCCA CU CGUG‐3’) were synthesized from GenePharma. After 24 h incubation, the cells were transfected with siRNA and miRNA using lipofectamine 3000 (Invitrogen, Carlsbad, CA), respectively.

### 2.4 RT‐qPCR

Total RNAs were extracted through Trizol (Thermo Fisher, USA) and reversed transcribed into cDNA. QPCR was performed using QuantiTect SYBR Green PCR Kit (Qiagen, Germantown, USA) by CFX96 PCR System (Thermo Fisher, USA). The primer sequence was showed as follows: GAS 5, F: 5′‐TCTATGACCTGGAAGC‐3′; R: 5′‐ATCTGATGGAACCCTA‐3′; miR‐21, F: 5′‐GCCGCTAGCTTATCAGACT‐3′; R: 5′‐AGTGCAGGGTCCGAGGTA‐3′; GAPDH, F: 5′‐ACAACTTTGGTATCGTG GAAGG‐3′; R: 5′‐GCCATCACGCCACAGTTTC‐3′; U6, F: 5′‐TGCGGGTGCTCG CTTCGGCAGC‐3′; R: 5′‐GTGCAGGGTCCGAGGT‐3′. 2^−ΔΔCT^ method to calculate the ratio of the relative expression of target genes*. Gapdh* was used as an internal control for lncRNA GAS5, and U6 for miRNAs. Fold changes were calculated by the $2^{-\Delta \Delta C_{t}}$ method.

### 2.5 Western blot analysis assay

Proteins were collected from HCMs cells through protein lysis buffer (Solarbio) and quantified by a BCA kit (Solarbio). Proteins (80 μg) were electrophoresed via 10% SDS‐PAGE gel and transferred to the NC membrane (Millipore). Then, the membrane was blocked by 5% nonfat milk (Solarbio) for 1 h at room temperature, and then incubated with the corresponding primary antibodies overnight at 4°C. The next day, membranes were washed 3 times with 0.1% TBST buffer and then incubated with HRP‐conjugated secondary antibodies for 1 h at room temperature. Primary antibodies were shown as follows: PTEN (1:1000; ab31392, Abcam), β‐actin (1:5000; ab8227, Abcam). The blot signals were detected by the ECL kit (Pierce), and the intensity was quantified via Image J 6.0 (Bio‐Rad).

### 2.6 Flow cytometry

First, HCM cells (1 × 10^6^) were washed twice with pre‐chilled PBS, followed by flow cytometry using the Annexin V Fluorescein (FITC) Apoptosis Detection Kit I (BD Biosciences; includes PI staining material). Among them, Annexin V‐positive cells indicate early apoptotic cells, propidium iodide (PI)‐positive cells indicate necrotic cells, and double‐positive cells indicate late apoptotic cells.

### 2.7 Caspase‐3 activity detection

According to the instructions of the Caspase‐3 kit(cat: BC3830, Solarbio). After transfection for 48 h, HCM cells were lysed and transferred to 96‐well plate. Then, the a 96‐well plate was incubated at 37°C for 2 h, the OD value was detected at 405 nm by the microplate reader.

### 2.8 Methyl thiazolyl tetrazolium (MTT) assay

HCMs (5 × 10^3^ cells/well) were cultured in 96‐well plates. The HCMs were exposed to different treatments, following incubated with MTT reagent (10 μL) for 4 h. 4 h later, removing the medium and adding DMSO (150 μL/well) for 10 min to dissolve formazan crystals. The cell viability was detected at 550 nm using a microplate reader (Bio‐Tek).

### 2.9 Cell migration and invasion assay

For migration assay, in the upper chamber of the Corning chamber, HCMs were cultured in RPMI‐1640 medium without 10% FBS. Add medium containing 10% FBS to the lower chamber. After the transfection of cells, they were maintained under normal or hypoxic conditions for 48 h. The migrated cells were subsequently fixed with paraformaldehyde followed by crystal violet staining. For cell invasion, a polycarbonate Transwell filter pre‐coated with Matrigel (BD Bioscience) was pressed on the culture plate in advance, and then only RPMI‐1640 medium was added to the upper layer, and the lower layer was Complete medium supplemented with 10% FBS is required. After the cells were transfected and cultured under normal or hypoxic conditions for 48 h, the free cells on the upper membrane were wiped off with a cotton swab, and then the invaded cells in Matrigel were fixed with 20% methanol, and stained with 5% crystal violet to observe.

### 2.10 Luciferase reporter assay

Bioinformatics software (https://cm.jefferson.edu/rna22/Precomputed/) was used to predict potential binding sites between lncRNA GAS5 and PTEN. First, the sequences of Wt‐GAS5/mut‐GAS5 and wt‐PTEN 3'UTR/mut‐PTEN 3'UTR need to be amplified, respectively, and then inserted into the pGL3 vector, respectively. Plasmids (0.5 µg; 20 pmol/µL) of successful WT or mutant 3'‐UTR DNA sequences were constructed for transfection. For luciferase assay experiments, HCM or HEK293 cells were first seeded and cultured in 48‐well plates and cotransfected with 50 nM miR‐21 and 0.2 µg luciferase reporter plasmid using Lipofectamine 3000 (Invitrogen). Forty‐eight hours after transfection, luciferase activity was detected using the Dual Luciferase Reporter Assay System (Promega).

### 2.11 Data analysis

All values are expressed as mean ± standard deviation (SD), and data were analyzed using one‐way analysis of variance, Fisher's least significant difference t test was used for pairwise comparison or Student't‐test. *p* < .05 was considered to have a significant difference.

## 3 RESULTS

### 3.1 Upregulation of GAS 5 and PTEN expression and downregulation of miR‐21 expression are correlated in hypoxic HCM

**Figure 1:**
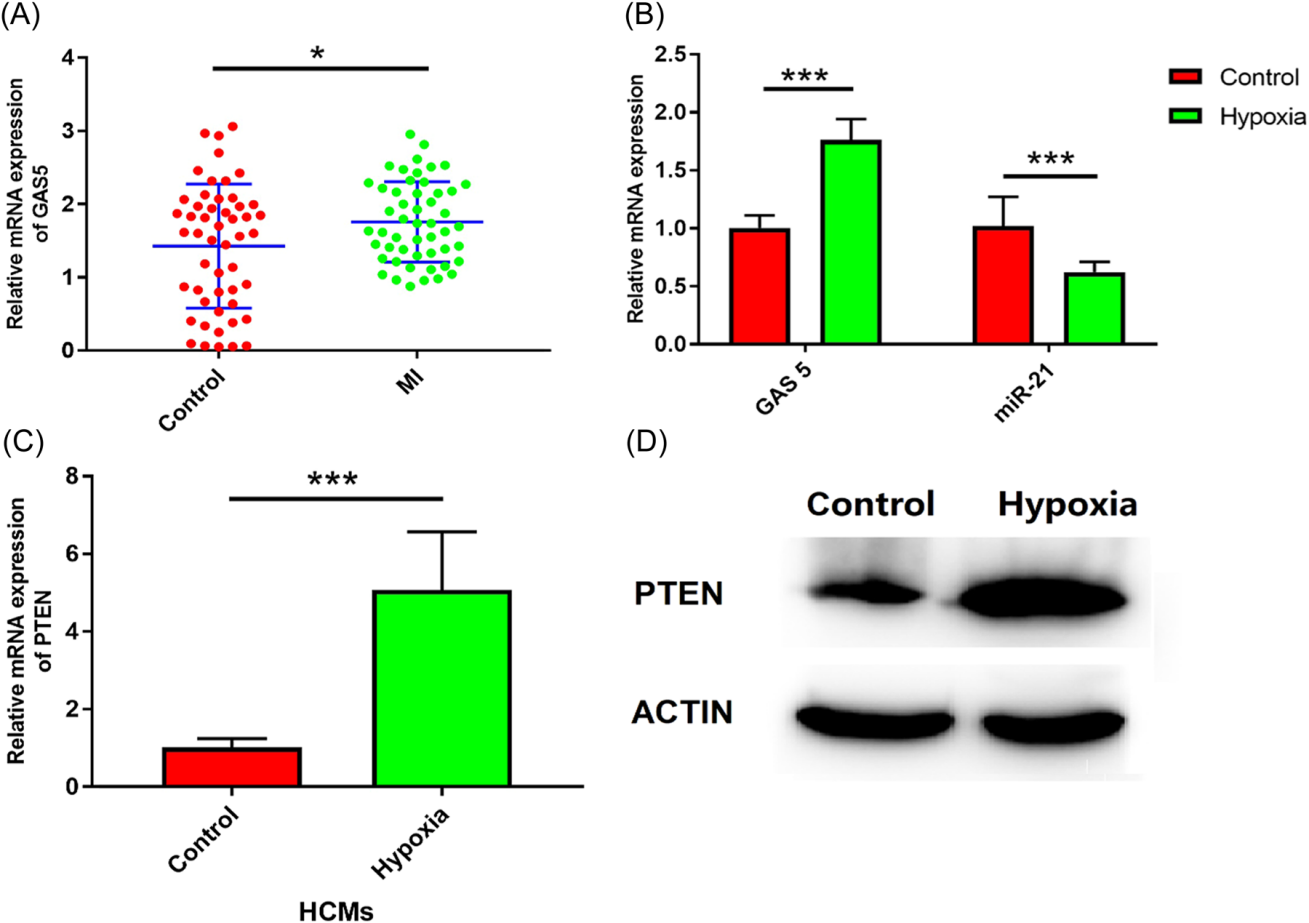
Hypoxia induced the increase of GAS 5 and PTEN but the reduction of miR‐21 in human cardiomyocytes. (A) GAS 5 expression was detected from peripheral blood collected from patients with myocardial infarction (MI) and controls. *n* = 51; Error bar, mean ± SD. ***p < .001; two‐tailed *t*‐test. (B) HCMs were cultured for 24 h to induced hypoxia injury under hypoxia condition. Then, GAS 5 (*n* = 3) and miR‐21 (*n* = 3) expression were quantified by qRT‐PCR. Error bar, mean ± SD. ***p < .001; two‐tailed *t*‐test. (C, D) PTEN expression was respectively determined by western blot analysis and qRT‐PCR in HCMs under hypoxia condition. *n* = 3; ***p < .001; two‐tailed *t*‐test.

First, we collected peripheral blood samples from patients with MI. We found that GAS 5 was increased in MI patients compared to the controls (Figure 1A, *p* < .05). Consistent with this result, GAS5 was remarkably increased in HCMs under hypoxia (Figure 1B, *p* < .001). Moreover, a previous study has reported that miR‐21 was dysregulated in hypoxia.^18^ Our result demonstrated that miR‐21 was significantly reduction in hypoxic HCMs (Figure 1B, *p* < .001). In contrast, PTEN was remarkably increased in HCMs under hypoxia conditions (Figures 1C,D, *p* < .001).^11^

### 3.2 Downregulation of GAS 5 expression attenuated hypoxia‐induced cell injury

**Figure 2:**
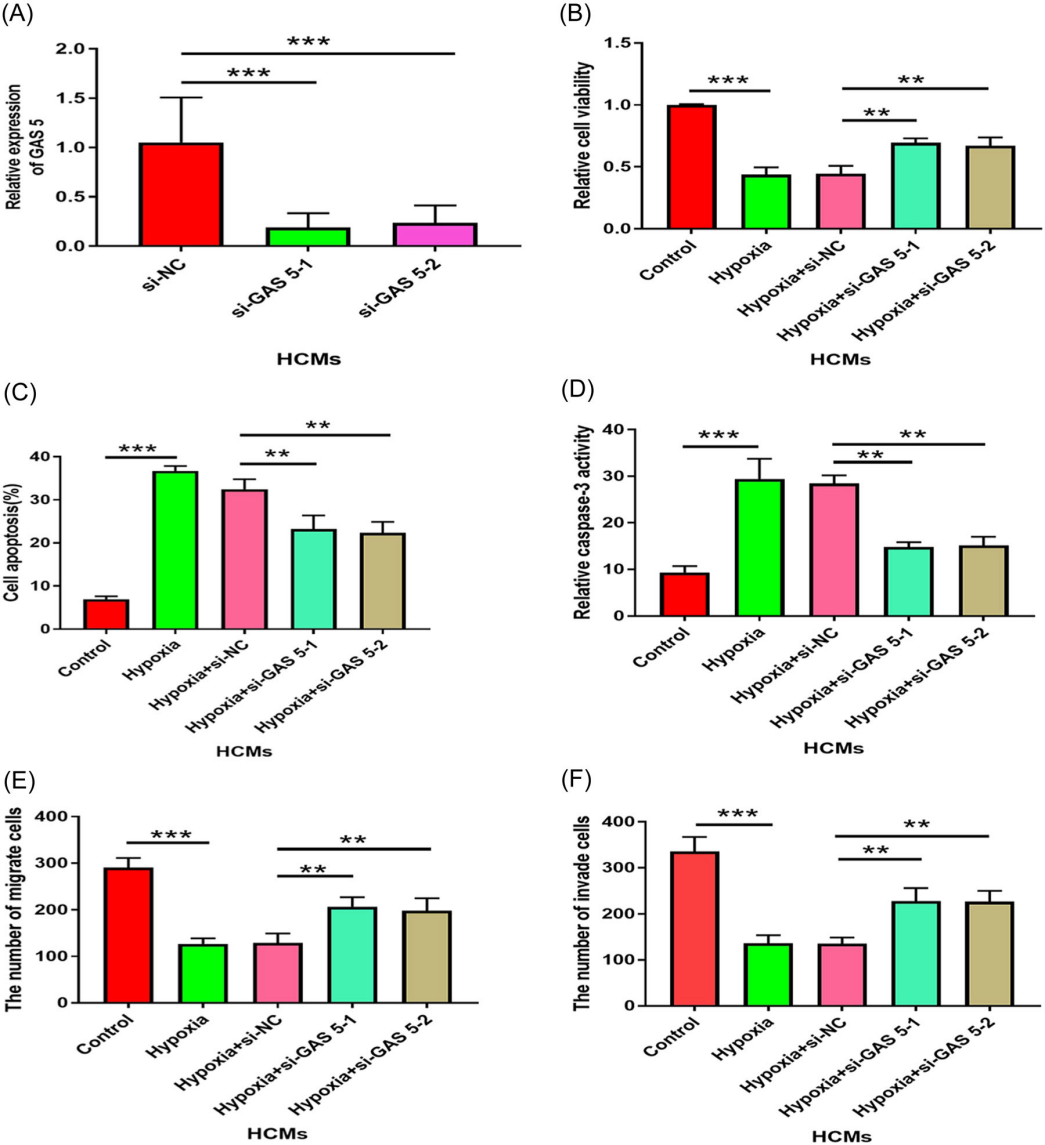
GAS 5 knockdown attenuated hypoxia‐induced cell injuries in HCMs. (A) GAS 5 expression was quantified by qRT‐PCR in HCMs after transfection with GAS 5 siRNAs (si‐GAS5‐1 and si‐GAS 5‐2) or negative control (si‐NC). *n* = 3; ***p < .001; Data were analyzed by One‐way ANOVA. The pairwise comparison after one‐way ANOVA analysis was analyzed by LSD‐t. (B) Cell viability was detected from different treatment group through MTT assay, such as control HCMs, hypoxic HCMs, and hypoxic HCMs transfected with si‐NC or GAS 5 siRNAs (si‐GAS 5‐1 and si‐GAS 5‐2). n = 3; **p < .01; ***p < .001; Data were analyzed by One‐way ANOVA. The pairwise comparison after one‐way ANOVA analysis was analyzed by LSD‐t. (C, D) Cell apoptosis and caspase‐3 activity were detected in control HCMs, hypoxic HCMs, and hypoxic HCMs transfected with si‐NC or GAS 5 siRNAs (si‐GAS 5‐1 and si‐GAS 5‐2). *n* = 3; **p < .01; ***p < .001; Data were analyzed by One‐way ANOVA. The pairwise comparison after one‐way ANOVA analysis was analyzed by LSD‐t. (E, F) Cell migration and invasion of control HCMs, hypoxic HCMs, and hypoxic HCMs transfected with si‐NC or GAS 5 siRNAs (si‐GAS 5‐1 and si‐GAS 5‐2) were quantififed by Transwell assay. *n* = 6; **p < .01; ***p < .001; Data were analyzed by One‐way ANOVA. The pairwise comparison after one‐way ANOVA analysis was analyzed by LSD‐t. ANOVA, analysis of variance; LSD‐t, least significant difference t test; MTT, methyl thiazolyl tetrazolium.

A previous report has proved that hypoxia can induce cell injury. Therefore, we want to explore whether GAS5 could affect cell injury under hypoxia. Firstly, we knockdown the endogenous GAS5 in HCMs by siRNA transfection. Our result indicated that GAS 5 expression was significantly decreased after transfection of siRNA (Figure 2A, *p* < .001). Moreover, hypoxia significantly reduces cell viability (Figure 2B, *p* < .001), promotes apoptosis (Figure 2C, *p* < .001), and activates caspase‐3 (Figure 2D, *p* < .001), while knocking down GAS 5 rescued these events in hypoxia (Figure 2). Meanwhile, knocking down GAS 5 prevented the hypoxia‐ mediated inhibition effect of cell migration (Figure 2E, *p* < .01) and invasion (Figure 2F, *p* < .01). Overall, these results indicated that knocking down GAS 5 attenuated the cell injury by hypoxia‐induced.

### 3.3 MiR‐21 is the direct target of the GAS 5

**Figure 3:**
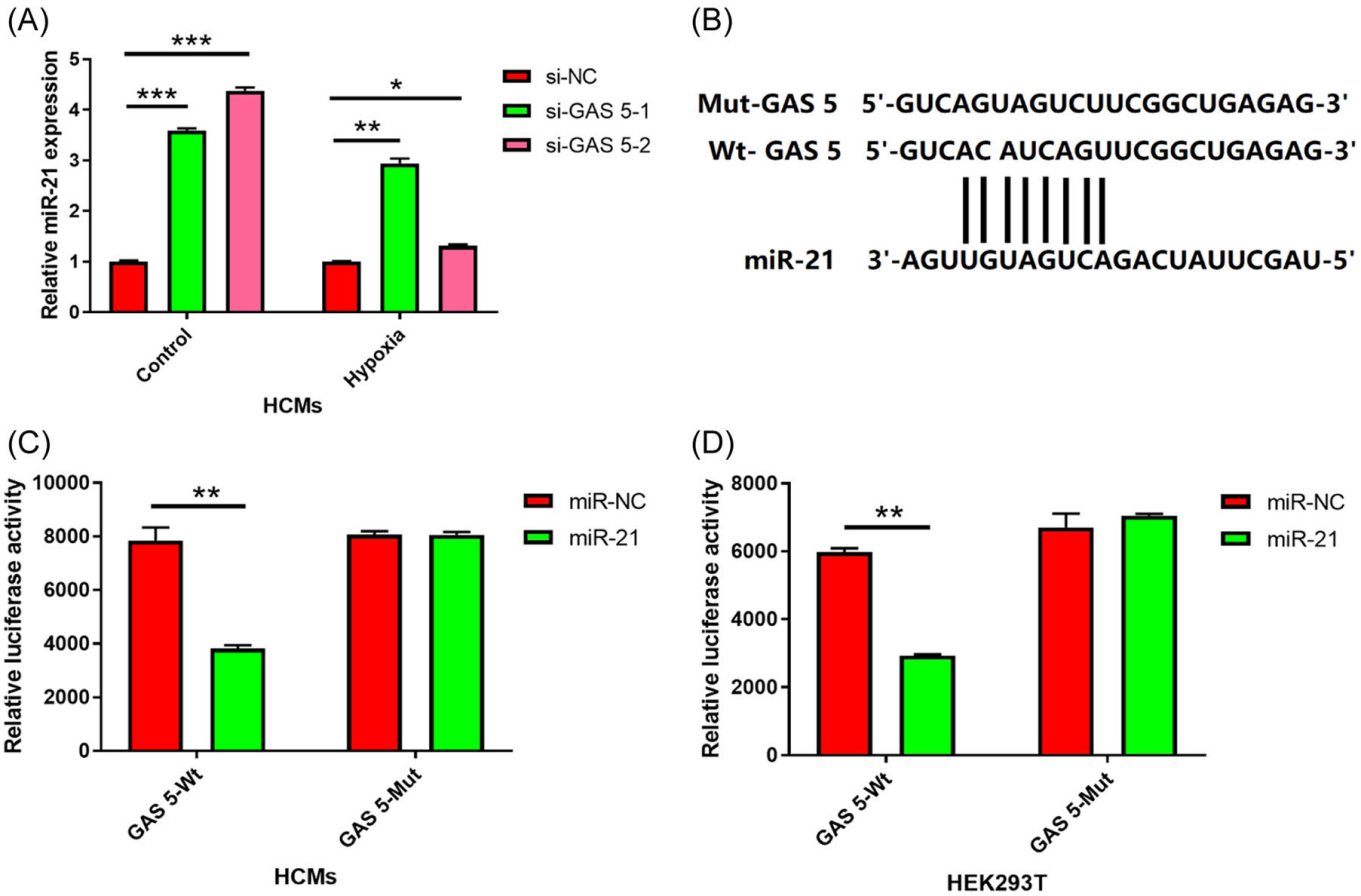
GAS 5 negatively regulated miR‐21. (A) Relative miR‐21 expression in the control or hypoxic HCMs transfected with GAS 5 siRNAs (si‐GAS 5‐1 and si‐GAS 5‐2) or negative control (si‐NC) was quantified by qRT‐PCR. *n* = 3; *p < .05; **p < .01; ***p < .001; Data were analyzed by One‐way ANOVA. The pairwise comparison after one‐way ANOVA analysis was analyzed by LSD‐t. (B) The prediction for miR‐21 binding sites on GAS 5 transcript and schematic of luciferase reporter vector constructs GAS 5 wild‐type (GAS 5 Wt) and the miR‐21‐binding‐site mutated (GAS 5 Mut) one. (C, D) The luciferase activities in HCMs and HEK293T cells cotransfected with miR‐21 or miR‐NC mimics and luciferase reporters containing GAS 5 Wt or GAS 5 Mut. *n* = 3; **p < .01; two‐tailed *t*‐test. ANOVA, analysis of variance; LSD‐t, least significant difference t test.

We utilized online software miRcode (http://www.mircode.org/mircode/) to predict the miRNA target of GAS5. It is predicted that miR‐21 is a candidate miRNA for GAS5 (Figure 3B). Under the condition of hypoxic, knocking down GAS5 obviously increased the miR‐21 expression in HCMs, revealing GAS5 negatively regulated miR‐21 expression (Figure 3A, *p* < .01). To further verify whether GAS5 functionally interacts with miR‐21, we used a dual luciferase reporter system. The wild type (WT) GAS5 or mutant was cloned into the pGL3.1‐basic vector and cotransfected with miR‐21 mimic or control into HCMs. We found that miR‐21 expression obviously downregulated the luciferase activity of WT but not mutated GAS5 (Figure 3C, *p* < .01). Similar observations were performed with HEK293 cells (Figure 3D, *p* < .01). Overall, miR‐21 is the direct target of the GAS5.

### 3.4 MiR‐21 could negatively mediate PTEN expression

**Figure 4:**
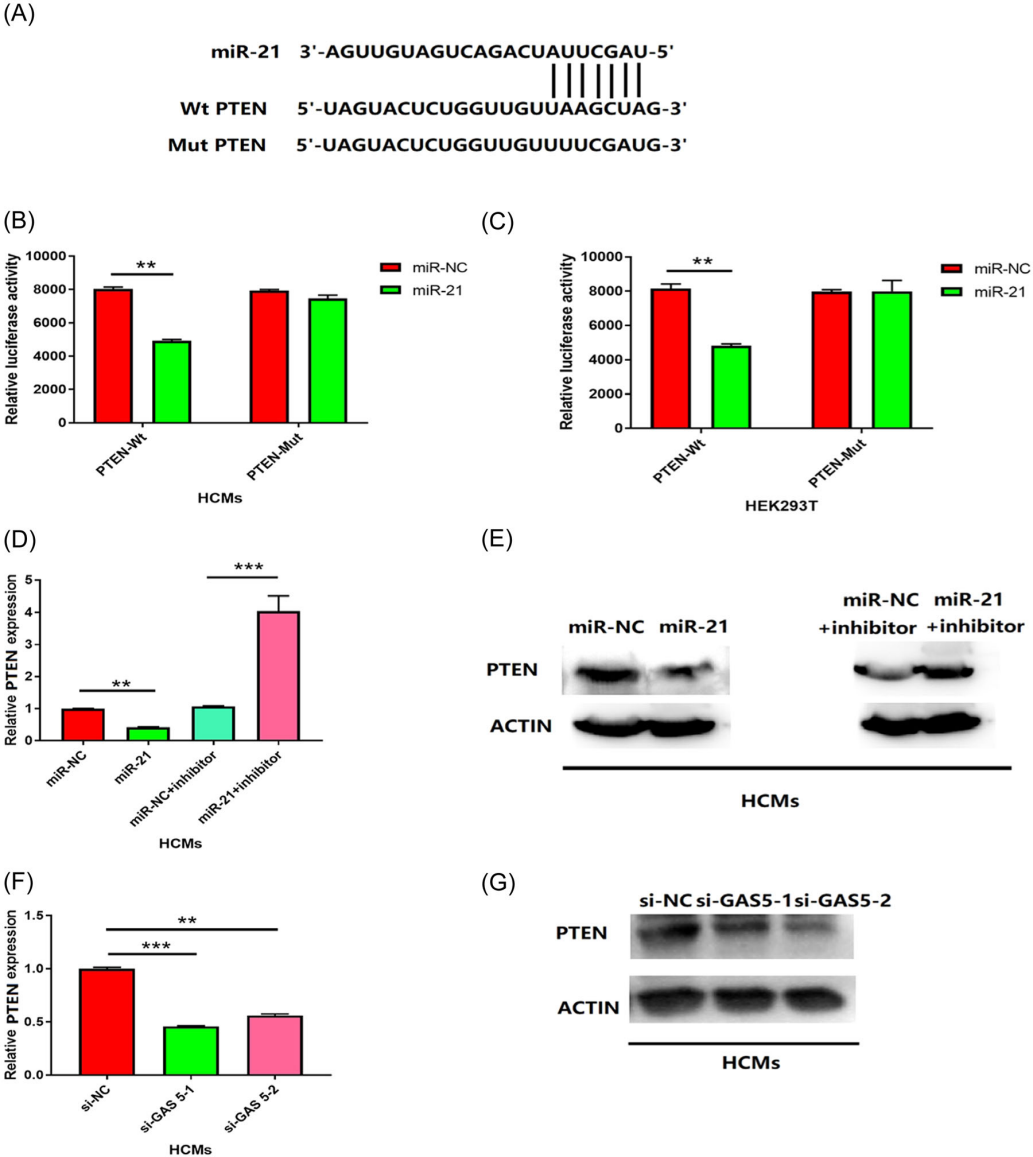
PTEN was a target of miR‐21. (A) Schematic of predicted wild‐type (PTEN 3′ UTR (Wt)) or mutated (PTEN 3′ UTR(Mut)) miR‐21 binding sites in the 3′ UTR of PTEN. (B, C) Relative luciferase activities of plasmids carrying wide‐type (PTEN 3′ UTR(Wt)) or mutant (PTEN 3′ UTR(Mut)) PTEN 3′ UTR in HCMs and HEK293T cells cotransfected with miR‐21 mimics or miR‐NC. *n* = 3; **p < .01; two‐tailed *t*‐test. (D, E) PTEN expression in HCMs transfected with indicated microRNA mimics and microRNA inhibitors or their respective negative controls were detected by qRT‐PCR and western blot. *n* = 3; **p < .01; ***p < .001; Data were analyzed by One‐way ANOVA. The pairwise comparison after one‐way ANOVA analysis was analyzed by LSD‐t. (F, G) PTEN expression in HCMs transfected with GAS 5 siRNAs (si‐GAS 5‐1 and si‐GAS 5‐2) or negative control (si‐NC) were measured by qRT‐PCR and western blot. n = 3; **p < .01; ***p < .001; Data were analyzed by One‐way ANOVA. The pairwise comparison after one‐way ANOVA analysis was analyzed by LSD‐t. ANOVA, analysis of variance; LSD‐t, least significant difference t test.

A previous study reported that miRNA could regulate gene expression by combining with the sequence of 3′‐UTR.^19^ Since GAS 5 targets miR‐21, we also predicts the target of miR‐21. We found that miR‐21 could bind to the 3′ UTR of PTEN (Figure 4A). Dual‐luciferase reporter assay was used to further confirm. WT‐3′ UTR/Mut‐3′ UTR of PTEN binding site of miR‐21 was inserted into the reporter vector and cotransfected with miR‐21 or control miRNA into HCMs. We found that the expression of miR‐21 has remarkably reduced the luciferase activity of WT but not mutant (Figure 4B, *p* < .01). Similar results were performed with HEK293 cells (Figure 4C, *p* < .01). Since miR‐21 targeted PTEN, we also detect whether miR‐21 can regulate the expression of PTEN. MiR‐21 overexpression in HCMs obviously decreased the PTEN mRNA and protein expression (Figures 4D,E, *p* < .001). In contrast, suppression of miR‐21 overexpression caused the elevation of the PTEN expression (Figures 4F,G, *p* < .001). Thence, we found that miR‐21 could negatively regulate PTEN expression. Since GAS 5 targets the miR‐21, we further investigated whether GAS 5 regulated PTEN expression. Knocking down GAS 5 significantly reduced the PTEN expression. Overall, these data indicated that PTEN was regulated by GAS5/miR‐21.

### 3.5 GAS 5/miR‐21/PTEN axis was associated with hypoxia‐induced cell injury in HCMs

**Figure 5:**
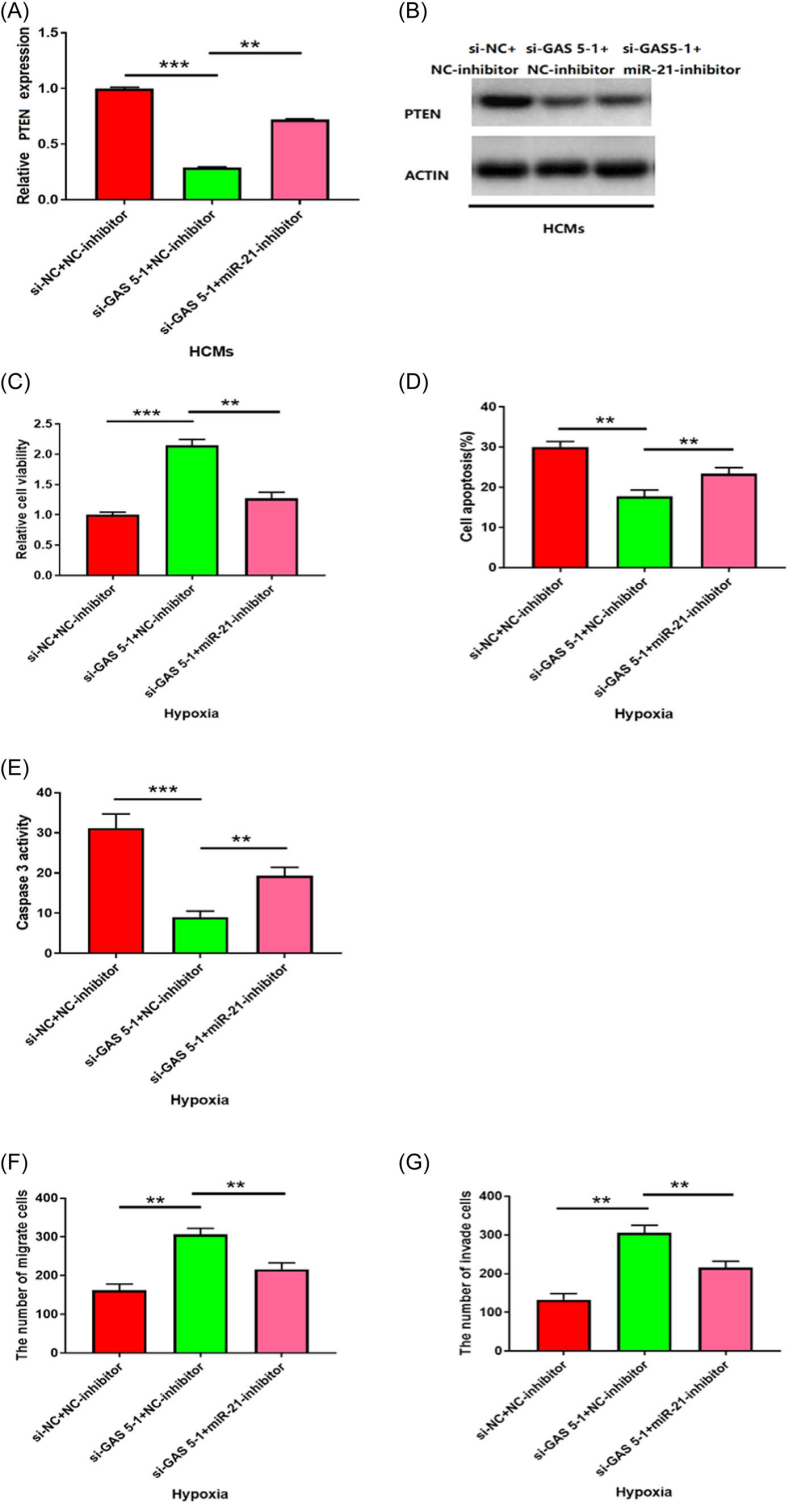
GAS 5/miR‐21/PTEN axis regulated hypoxia‐induced cell injuries in HCMs. (A, B) Relative mRNA and protein levels of PTEN in HCMs cotransfected with siRNA negative control and negative control inhibitor (si‐NC + NC‐inhibitor), GAS 5 siRNA and negative control inhibitor (si‐GAS 5‐1 + NC‐inhibitor), or GAS 5 siRNA and miR‐21 inhibitor (si‐GAS 5‐1 + miR‐21‐ inhibitor) were measured by qRT‐PCR and western blot. *n* = 3; **p < .01; ***p < .001; Data were analyzed by One‐way ANOVA. The pairwise comparison after one‐way ANOVA analysis was analyzed by LSD‐t. (C) Inhibition of miR‐21 decreased the enhanced cell viability of hypoxic HCMs due to knockdown of GAS 5 through MTT assay. *n* = 3; **p < .01; ***p < .001; Data were analyzed by One‐way ANOVA. The pairwise comparison after one‐way ANOVA analysis was analyzed by LSD‐t. (D, E) Inhibition of miR‐21 increased the decreased cell apoptosis of hypoxic HCMs due to knockdown of GAS 5 through Flow cytometry and caspase‐3 activity assay. *n* = 3; **p < .01; ***p < .001; Data were analyzed by One‐way ANOVA. The pairwise comparison after one‐way ANOVA analysis was analyzed by LSD‐t. (F, G) Inhibition of miR‐21 suppressed the increased migration and invasion of hypoxic HCMs due to knockdown of GAS 5 through Transwell assay. *n* = 6; **p < .01; Data were analyzed by One‐way ANOVA. The pairwise comparison after one‐way ANOVA analysis was analyzed by LSD‐t. ANOVA, analysis of variance; LSD‐t, least significant difference t test; MTT, methyl thiazolyl tetrazolium.

According to the above results, we investigated their functions in hypoxia. Knocking down GAS 5 could down‐regulate the transcription of PTEN, while PTEN downregulation by GAS5 knocking down depended on miR‐21, as inhibition of the miR‐21 rescued the downregulation the transcription of PTEN (Figure 5A, *p* < .01). Moreover, knocking down GAS5 also remarkably decreased the protein expression of PTEN and inhibition of miR‐21 prevented the PTEN protein expression downregulation by GAS 5 knocking down (Figure 5B, *p* < .001). Knocking down GAS 5 obviously increased cell viability, cell migration, and invasion (Figures 5C,F,G, *p* < .001), decreased cell apoptosis (Figure 5D, *p* < .001), and caspase‐3 activity (Figure 5E, *p* < .001) in hypoxia. MiR‐21 downregulation could reduce these effects by GAS5 knocking down‐induced. Overall, our results revealed that the GAS 5/miR‐21/PTEN axis could regulate hypoxia‐induced cell injury in HCMs.

## 4 DISCUSSION

MI is a severe heart disease, which eventually leads to heart failure. Although the prevention and treatment of MI have been promoted, whereas the deaths rates of MI still maintain a high level in the world. Therefore, there is an urgent to understand the molecular mechanisms of MI and find the targets to treat MI.

A growing number of lncRNAs have been linked to various kinds of cardiovascular diseases. For instance, increased expression of lncRNA Kcna2 Antisense RNA (Kcna2 AS) led to an increased incidence of ventricular arrhythmias in association with heart failure.^20^ LncRNA UCA1 was able to promote the progression of cardiac hypertrophy, a condition associated with a series of cardiovascular diseases, including heart failure.^21^ In this study, we explored the functional role of lncRNA GAS5, a widely reported tumor suppressive gene,^22^, ^23^, ^24^ in hypoxia‐injured H9c2 cells, aiming to evaluate the importance of lncRNA GAS5 in heart failure caused by MI. According to reports, hypoxia (hypoxic tension) stress can cause many pathological reactions, such as infarction. Under hypoxic conditions, cell apoptosis will increase, and it will also increase the remodeling of a myocardial structure.^25^ In our study, we confirmed the increased apoptosis of HCM and increased caspase‐3 activity under hypoxic conditions. Meanwhile, we also observed that the cell viability, migration, and invasion capabilities of HCMs have been significantly reduced.

LncRNAs are critical factors in regulating cell activities, including cell proliferation, apoptosis, and metabolism.^26^ Growing evidence has proved that lncRNAs were regulated the development, progression, and diagnosis of diseases.^27^, ^28^, ^29^ The function of lncRNAs in cardiovascular disease has been reported.^30^, ^31^, ^32^ LncRNA CHRF has been reported that it was involved in cardiac hypertrophy by regulating miR‐489.^33^ LncRNA GAS5 was first studied in various cancers and serves as a tumor suppressor gene.^34^, ^35^, ^36^ To date, the function of GAS 5 still remained unclear in hypoxia and MI. Therefore, we investigated the function of GAS5 in cardiomyocyte injury by hypoxia‐induced. In our study, we first identified that lncRNA GAS5 was associated with MI. Meanwhile, GAS5 expression was also increased in HCMs by hypoxia‐induced. These results revealed the potential effect of GAS5 in MI. In our study, knocking down GAS5 weakened the injury of cell by hypoxia‐induced and increased, the cell viability, reduced apoptosis and rescued the cell's ability to migrate and invasion. Overall, these data indicated the function of GAS5 in hypoxia‐induced HCMs injury.

The target‐mimic, sponge/decoy function of lncRNA on miRNAs has been reported.^37^ The miR‐21, an abundant miRNA in cardiomyocytes, shows a cardioprotective effect in MI process,^38^ while the underlying mechanism is less clear. Studies showed that miR‐21 could functionally interact with lncRNAs. For example, lncRNA BISPR stimulated progression of thyroid papillary carcinoma by regulating miR‐21‐5p and lncRNA MEG3 suppressed proliferation of chronic myeloid leukemia cells by sponging miR‐21.^39^ It was previously demonstrated that GAS5 and miR‐21 formed a reciprocal repression feedback loop and GAS5 negatively regulated miR‐21 expression through the RISC.^40^ Moreover, GAS5 regulated proliferation and apoptosis in the growth plate by modulating fibroblast growth factor 1 (FGF1) expression via mediating miR‐21.^41^ In addition to heart disorders, the interaction of GAS5 and miR‐21 has been found in osteoarthritis, atherosclerosis and cancer, etc.^42^, ^43^, ^44^ In our study, we found that miR‐21 was the target of GAS5 and miR‐21 targeted PTEN. In addition, we also identified that miR‐21 expression was decreased in HCMs under hypoxia, which was associated with cell viability, migration and invasion in HCMs by hypoxia‐induced. This result demonstrated that miR‐21 was positively the cell growth and invasion in HCMs. Although we found the correlation with PTEN and hypoxia, the precise function of PTEN in hypoxia need to be further investigated.

Of course, our research also has limitations. For example, (1) The number of clinical samples is still relatively small, and more samples are needed. (2) We need to construct an animal model to confirm the results of our in vitro experiments through in vivo research. (3) Our detection indicators still need to be closer to clinical applications, such as identifying specific target sites that can provide reference for future drug design.

In conclusion, our results indicate that GAS5 can regulate the process of MI by targeting the miR‐21/PTEN axis. Therefore, both GAS5 and miR‐21/PTEN may become key targets for the treatment of MI in the future.

## AUTHOR CONTRIBUTIONS

Qianli Wang and Zan Xie designed the study and supervised the data collection. Qianli Wang and Zan Xie analyzed the data and interpreted the data. Zan Xie prepare the manuscript for publication and reviewed the draft of the manuscript. Qianli Wang and Zan Xie revised the manuscript. All authors have read and approved the manuscript.

## CONFLICT OF INTEREST STATEMENT

The authors declare no conflict of interest.

## Data Availability

We will provide raw data upon reasonable request.
